# An Exception to the Rule: Carry-Over Effects Do Not Accumulate in a Long-Distance Migratory Bird

**DOI:** 10.1371/journal.pone.0086588

**Published:** 2014-02-11

**Authors:** Nathan R. Senner, Wesley M. Hochachka, James W. Fox, Vsevolod Afanasyev

**Affiliations:** 1 Cornell Lab of Ornithology, Cornell University, Ithaca, New York, United States of America; 2 British Antarctic Survey, Cambridge, United Kingdom; Institute of Ecology, Germany

## Abstract

Recent years have seen a growing consensus that events during one part of an animal's annual cycle can detrimentally affect its future fitness. Notably, migratory species have been shown to commonly display such carry-over effects, facing severe time constraints and physiological stresses that can influence events across seasons. However, to date, no study has examined a full annual cycle to determine when these carry-over effects arise and how long they persist within and across years. Understanding when carry-over effects are created and how they persist is critical to identifying those periods and geographic locations that constrain the annual cycle of a population and determining how selection is acting upon individuals throughout the entire year. Using three consecutive years of migration tracks and four consecutive years of breeding success data, we tested whether carry-over effects in the form of timing deviations during one migratory segment of the annual cycle represent fitness costs that persist or accumulate across the annual cycle for a long-distance migratory bird, the Hudsonian godwit, *Limosa haemastica*. We found that individual godwits could migrate progressively later than population mean over the course of an entire migration period, especially southbound migration, but that these deviations did not accumulate across the entire year and were not consistently detected among individuals across years. Furthermore, neither the accumulation of lateness during previous portions of the annual cycle nor arrival date at the breeding grounds resulted in individuals suffering reductions in their breeding success or survival. Given their extreme life history, such a lack of carry-over effects suggests that strong selection exists on godwits at each stage of the annual cycle and that carry-over effects may not be able to persist in such a system, but also emphasizes that high-quality stopover and wintering sites are critical to the maintenance of long-distance migratory populations.

## Introduction

Migratory species, and especially those breeding at Arctic and sub-arctic latitudes, face severe time constraints during their annual cycles [1]. Many species must properly time their annual activities to correspond with resource peaks at disparate sites spread widely across the globe [2]. Migrants frequently incur significant physiological costs — from lowered immune function [3] to increased levels of oxidative damage [4] — during the course of their migratory movements and breeding and this stress can necessitate trade-offs between allocating resources to current and future needs [5]. There is an increasing recognition that these trade-offs not only have short-term consequences, but also can carry over into future seasons and influence events that were previously believed to be disconnected [6]. Ultimately these carry-over effects can even affect population dynamics [7].

Carry-over effects have been documented in an array of species, not only long-lived migratory birds and ungulates, but also shorter-lived, largely sedentary organisms like reptiles, fish, and invertebrates [5]. Most studies have investigated how events occurring during the nonbreeding season can alter the future breeding success of individuals. For instance American redstarts, *Setophaga ruticilla*, occupying lower-quality habitats during winter departed for and arrived on their breeding grounds later and had reduced breeding success [8], [9]. Similar findings in Icelandic black-tailed godwits, *Limosa limosa islandica*, and sockeye salmon, *Oncorhynchus nerka*, suggest that such effects are common across migratory taxa [10], [11]. In general, delaying the initiation of an annual-cycle event, such as migration or moult, is the most commonly identified manifestation of carry-over effects [5].

To date, and largely as a result of the continued difficulty in tracking individual migrants, there has been relatively little effort to identify how carry-over effects are manifested during the nonbreeding season or whether they persist or even accumulate over sequential life-history phases [12], [13]. This leads to the question: When during the annual cycle do carry-over effects arise and when do they disappear? It is possible that once carry-over effects have been incurred, they never disappear; conversely there may be mechanisms built into the annual cycle that reduce or even erase residual stress, limiting the persistence of carry-over effects. Identifying which of these alternatives actually occurs is key to understanding the selection pressures acting upon individuals throughout their annual cycles, and thus is critical for prioritizing conservation actions [14], [15].

Here we present results from a study in which we examine the accumulation and dissipation of carry-over effects across an entire annual cycle, using data from 26 adult Hudsonian godwits (hereafter, godwits), *Limosa haemastica*, carrying British Antarctic Survey geolocation-tracking devices; these data come from three consecutive years of migration coupled with four consecutive years of data on breeding success. Godwits migrate the entire length of the Western Hemisphere and must breed within a short, nine-week sub-arctic summer, meaning that their annual cycle is likely severely time constrained and thus increasing the likelihood that carry-over effects on timing of events such as nesting will have detrimental consequences [16]. Therefore, we predict that those godwits falling behind during one portion of their annual cycle will either not recoup this lost time or subsequently fall further behind, resulting in a late arrival on the breeding grounds, a failure to nest during the narrow phenological peak for breeding, and reduced breeding success [1].

## Materials and Methods

### Ethics Statement

Our study was approved by the Institutional Animal Care and Use Committee at Cornell University (Application #2001-0051) and carried out in accordance with their animal care guidelines. Handling of birds and attachment of loggers was carried out under Migratory Bird Banding Permit #20022, Federal Collecting Permit #MB-150573, Federal Fish and Wildlife Permit #MB789758-9, and Alaska State Permit #11-092. Permission from the ConocoPhillips Company is required to access our study site at Beluga River, Alaska (61.21°N, 151.03°W); approval can be obtained by contacting the environmental field office of the ConocoPhillips Company at 907-776-2092. No endangered species were involved in this study.

### Field Methods

We studied breeding Hudsonian Godwits nesting at Beluga River, Alaska (61.21°N, 151.03°W) from 2009–2012. Nests were discovered using behavioral cues or by opportunistically flushing incubating individuals. We determined the number of nesting attempts made by an individual by monitoring each nest every 2–3 days until the nest had either failed or its eggs hatched. Replacement nests were found and monitored in the same manner as first nests. Upon hatching, all chicks in a brood were captured before leaving the nest and fitted with U.S. Geological Survey metal bands and unique alpha-alpha flags. Godwits fledge at the age of 28 days [17] and once the earliest chicks to hatch had potentially reached that age, we began surveying our study site daily for fledged chicks. We denoted an individual adult as having bred successfully if one or more chicks from its brood reached an age of at least 28 days.

Individual adult godwits were captured on nests and fitted with a U.S. Geological Survey metal band, a year-specific color band, and either a uniquely coded alpha-alpha flag (*n* = 27) or a British Antarctic Survey (BAS) Mk-14 or Mk-10 logger (*n* = 47) attached to a uniquely coded alpha-alpha flag. Mk-14 loggers weigh 1.4 g (2009) and Mk-10 loggers weigh 1.1 g (2010–2011), roughly 0.44–0.56% of mean godwit lean mass [17]. Logger-bearing flags were attached to the left upper tibia and separated from the tibio-tarsal joint by the color band to reduce potential wear on the joint. Returning individuals with loggers were recaptured on their nests and given new loggers to monitor the subsequent year's movements.

### Geolocation Data

After a logger's retrieval, its data were downloaded and initially processed using BAS software (version 8, March 2010). BAS loggers measure ambient light levels once per minute and record the highest level for each five-minute period from throughout the deployment of the logger. Light level information was transformed to identify the timing of sunrise and sunset for each day of deployment, which was, in turn, used to calculate the approximate location of the logger each day. There are limitations to the precision and accuracy of locations given by this method. As such, decision rules must be applied to the raw movement data so that errors caused by unusual light-exposure patterns are not confused with actual bird movements [18]. In this initial phase, we applied only one decision rule to each individual's movement data: all sunrises not preceded by 4 or more hours of darkness were excluded. In a second phase, we applied a filter developed for use with satellite movement data [19], which limits daily movements based on two criteria — redundant distance and maximum speed. Redundant distance refers to situations in which an individual is largely stationary and location readings on three consecutive days may have two locations in very close proximity to each other and one that is far-flung and likely in error; the redundant distance filter detects this third location by analyzing the data set as a three-day moving window, identifying if one location is not in close proximity to the other two. We limited individuals to a redundant distance of 100 km and a maximum speed of 100 km h^−1^.

Using these filtered movement data we identified arrival and departure dates for each stop for each individual. From these histories, we created year-specific population mean schedules with which we contrasted the movement history of each individual. We determined whether each individual departed from the breeding grounds earlier or later than the mean population departure date (hereafter “relative timing of departure”) and whether or not they became progressively earlier or later with each subsequent arrival or departure (hereafter “rate of change”) during the entire annual cycle (Table 1). Analyzing the relative lateness or earliness of an individual's movements in relation to the annual population mean allowed us to correct for inter-annual differences in population-level timing that might affect the timing of individuals (for instance because of inclement weather or social cues), as well as for potential individual-specific schedules [20]. We reset annual timing deviations and rates of change after arrival at the breeding grounds — thus each individual's “year” started with its departure from the breeding grounds — to enable us to compare the timing of movements among individuals within a year, regardless of their previous history within the study.

**Table 1:** Transformation of movement data of individual Hudsonian godwits into scores that reflect the accumulation or dissipation of lateness during their annual cycles.

	Beluga Departure	Saskatchewan Arrival	Saskatchewan Departure	Amazon Arrival
Population Mean	6 July	8 July	20 August	25 August
Individual HX (raw data)	7 July	11 July	17 August	21 August
HX (Step 1 — Relative Timing)	+1	+3	−3	−4
HX (Step 2 — Rate of Change)	+1	+2	−6	−1

The first line displays the population mean (2009, *n* = 15) timing of arrival and departure for three consecutive sites in the godwit annual cycle. The second line displays the dates of the movements between those sites for one individual godwit, “HX.” The third line displays the relative timing of HX's movements in relation to the population mean. In this case, HX departed Beluga on 7 Jul and the population mean departure was 6 Jul; thus HX departed Beluga 1 day later than the mean (+1). HX arrived in Saskatchewan 3 days later than the mean and thus has a score of +3. In the fourth line is the rate of change of HX's movements. This score reflects the timing of HX's movements both in relation to the population mean, but also in relation to the timing of its previous movements. HX left Beluga 1 day later than the mean and, also, arrived in Saskatchewan an additional two days later than the mean (three days later in total), giving scores of +1 and +2. However, it departed Saskatchewan 3 days earlier than the mean, thus giving it a score of −6 (+3 to −3). Values calculated in Step 1 allowed us to account for inter-annual differences in the movements of the entire population. Values calculated in Step 2 allowed us to determine if an individual's rate of change from mean timing was part of an individually consistent schedule (i.e., an individual always departing three days later than the mean) or whether they reflected an individual becoming increasingly later (or earlier) — a potential manifestation of the existence of carry-over effects.

It is important to note that the timing of one event was not directly included in our models. Departure date from the northward migration stopover site in the mid-continental United States was not included because, unlike other stopovers used during the annual cycle, there is no one “site” that exists in this region [17]. Instead godwits use a suite of small and large wetlands, many of them ephemeral, which change on a yearly basis and span from central Texas to central South Dakota. In any given year, depending on wind and groundwater conditions, individuals may move amongst a number of these during a single northward migration (N.R. Senner unpubl. data). As such, there is no clear departure date that can be identified and, furthermore, those individuals departing on a flight to the breeding grounds from further south in this region would have a different relative departure date than those leaving from further north. Instead we quantified how many stops each individual made during their northward migration and the average stopover duration across those stops. We believe that these two measures act as valid statistical proxies for departure date during this period.

### Statistical Analysis

We analyzed our data to examine both the potential causes for an individual's deviation from the mean population timing and rate of change of this deviation between each subsequent portion of the annual cycle. We did this by first creating a series of sequential linear mixed-effect models to identify those conditions that affected an individual's rate of change between departure and arrival from each stage of the annual cycle. Each model included as its fixed effects a set of variables representing the timing of those events that immediately precede it — for instance, the model for the timing of departure from the breeding grounds included its timing deviation and rate of change from its arrival at the breeding grounds, the number of nests an individual had, and its breeding success. If a fixed-effect variable explained a significant portion of the variation in one model, it was carried over to the next model — in the case of the model for the timing of departure from Beluga River, no fixed effect was significant and thus the model for the timing of arrival in Saskatchewan only included the variable for the timing of departure from Beluga. We included individual and year (although year was never a significant random effect; Tables 2,3) as random effects in all models and assessed their significance using the “lmerTest” package in Program R.

**Table 2:** Model and parameter estimates explaining the variance in rate of change in timing deviations exhibited by Hudsonian godwits during their annual cycle (2009–2012).

Model	Parameters	Random Effects			Fixed Effects			
		K	σ^2^	SD	Variable	β	SE	*t*
Departure from Beluga River	Prior Breeding Success + Prior # Nests	Ind.	2.57	1.60	Intercept	−5.23	3.05	−1.72
		Year	0.00	0.00	PBS	2.98	1.95	1.52
		Res.	32.91	5.74	P#N	3.34	1.99	1.68
Arrival in Sask.	Beluga River Departure	Ind.	1.60	1.27	Intercept	0.33	0.30	1.10
		Year	0.00	0.00	BRD	0.01	0.03	0.32
		Res.	1.34	1.16				
Sask. Depart.	Sask. Arrival	Ind.	3.47	1.86	Intercept	−0.15	1.09	−0.14
		Year	0.00	0.00	SAA	−0.13	0.67	−0.20
		Res.	53.82	0.34				
Arrival in Amazon	Sask. Depart.	Ind.	0.00	0.00	Intercept	0.10	0.39	0.25
		Year	0.00	0.00	SKD	−0.07	0.05	−1.28
		Res.	8.08	2.84				
Departure from Amazon	Amazon Arrival	**Ind.**	**45.15**	**6.72**	Intercept	0.31	1.70	0.18
		Year	0.00	0.00	AMA	−0.59	0.38	−1.53
		Res.	47.52	6.89				
Arrival in Buenos Aires	Amazon Departure	Ind.	1.7×10^−12^	1.3×10^−6^	Intercept	0.54	0.53	1.02
		Year	0.64	0.80	AMD	−0.02	0.03	−0.79
		Res.	3.41	1.85				
Departure from Buenos Aires	Arrival in Buenos Aires	Ind.	28.01	5.29	Intercept	0.76	1.79	0.43
		Year	0.00	0.00	ABA	−0.47	0.74	−0.63
		Res.	79.42	8.91				
Arrival in Chiloe	Buenos Aires Departure	Ind.	1.27	1.13	Intercept	−0.41	0.67	−0.61
		Year	0.00	0.00	BAD	−0.02	0.06	−0.34
		Res.	18.47	4.30				
Departure from Chiloe	Arrival in Chiloe	Ind.	36.34	6.03	Intercept	1.08	2.15	0.51
		Year	1.5×10^−14^	1.2×10^−7^	ACH	0.83	0.42	1.99
		Res.	115.15	10.73				
Arrival in North America	Chiloe Departure	Ind.	0.01	0.11	Intercept	0.15	0.31	0.48
		Year	0.23	0.48	CHD	−0.01	0.01	−1.18
		Res.	0.88	0.94				
Arrival in Beluga River	N.A Arrival + #Stops + Avg. Stop. Duration	Ind.	1.54	1.24	**Intercept**	**−8.39**	**2.23**	**−3.75**
		Year	0.02	0.15	NAA	−0.28	0.34	−0.82
		Res.	3.42	1.85	**Stops**	**2.07**	**0.56**	**3.73**
					**ASD**	**0.38**	**0.11**	**3.39**

Bold-font variance and t-statistic values were determined to be significant at *P*<0.05 (*n* = 26).

**Table 3:** Model and parameter estimates for models explaining the variance in the relative timing deviations exhibited by Hudsonian godwits during their annual cycle (2009–2012).

Model	Parameters	Random Effects			Fixed Effects			
		K	σ^2^	St. Dev.	Variable	β	SE	*t*
Departure from Beluga River	Prior Breeding Success + Prior # Nests	Ind.	2.57	1.60	Intercept	−5.23	3.05	−1.72
		Year	0.00	0.00	PBS	2.98	1.95	1.52
		Res.	32.91	5.74	P#N	3.34	1.98	1.68
Departure from Saskatchewan	Beluga River Departure	Ind.	4.92	2.22	Intercept	0.39	1.10	0.35
		Year	0.00	0.00	**BRD**	**0.76**	**0.17**	**4.40**
		Res.	52.07	7.22				
Departure from Amazon	BRD + Sask. Departure	**Ind.**	**44.60**	**6.68**	Intercept	−0.16	1.71	−0.10
		Year	0.00	0.00	BRD	0.11	0.24	0.45
		Res.	49.74	7.05	**SAD**	**1.02**	**0.16**	**6.21**
Departure from Buenos Aires	SAD + Amazon Departure	Ind.	36.64	6.05	Intercept	1.13	1.77	0.64
		Year	0.00	0.00	SAD	0.03	0.23	0.15
		Res.	68.20	8.26	**AMD**	**0.73**	**0.17**	**4.34**
Arrival in Chiloe	AMD + Buenos Aires Departure	Ind.	1.31	1.15	Intercept	−0.39	0.68	−0.57
		Year	0.00	0.00	AMD	−0.00	0.08	−0.01
		Res.	18.74	4.33	BAD	0.97	0.07	14.5
Departure from Chiloe	BAD + Arrival in Chiloe	**Ind.**	**3.54**	**1.88**	Intercept	−0.25	0.53	−0.46
		Year	0.00	0.00	BAD	−0.03	0.10	−0.33
		Res.	4.16	2.04	ACH	0.05	0.09	0.55
Arrival in Beluga River	Chiloé Departure + N.A. Arr. + #Stops + Avg. Stop. Duration	Ind.	1.24	1.11	Intercept	−8.05	2.10	−3.84
		Year	0.88	0.94	CHD	0.25	0.24	1.06
		Res.	2.80	1.67	NAA	0.39	0.23	1.69
					**Stops**	**2.19**	**0.51**	**4.33**
					**ASD**	**0.32**	**0.11**	**2.85**

Bold-font variance and t-statistic values were determined to be significant at *P*<0.05 (*n* = 26).

We similarly created sequential linear mixed-effect models to identify those conditions that affected the timing of an individual's migration relative to the mean throughout their annual cycle. However, because of the high colinearity between the relative timing of an individuals' consecutive departures and arrivals (i.e., departure from the breeding grounds and arrival in Saskatchewan), and because we found no evidence for variation in flight times among individuals, we chose to use only the relative timing of departures in our models during the southward migration period. Again, significant fixed effects were carried over to the next model in the sequence and individual and year were included as random effects in all models.

A final mixed-effect logistic regression model with a binomial error distribution combined the significant variables from both the rate of change and relative migratory timing analyses to test the potential effects of these factors on an individual's breeding success. (In 2012 we only monitored the breeding season until hatch and thus only the number of nesting attempts by an individual was included in our models for that year.) Individual and year were again included as random effects.

We also monitored return rates of both logger- and flag-carrying adults in subsequent years through daily observations at the breeding site and at adjacent feeding locations. Because we never recorded an individual returning after it was an absent for a year, we calculated return rates as the proportion of observed returning individuals versus the proportion of potentially returning individuals. To determine if carry-over effects might account for those individuals that did not return, we used a logistic regression to test if prior breeding success, number of nesting attempts, and accumulated lateness during the previous year affected the return rates of individuals carrying loggers. Similarly, we used a logistic mixed-effect model for all banded adults containing prior breeding success, number of nesting attempts, and whether or not an individual was carrying a logger as fixed effects and individual and year as random effects to determine if either prior breeding success or the number of nesting attempts affected return rates in the wider banded population.

## Results

We deployed 79 geolocation tracking devices (hereafter, “loggers”) on 47 individual godwits from 2009–2011. We recovered loggers from 29 individuals (62%), yielding 43 complete tracks and 13 partial tracks (from loggers that failed during migration) from 26 individuals. (The loggers for 3 individuals failed within days of deployment and yielded no movement data.) Eleven individuals were tracked for three consecutive years (but only 9 individuals provided complete tracks for all three years), 6 additional individuals were tracked for two consecutive years, and the remaining 12 individuals were tracked for only one year.

**Figure 1:**
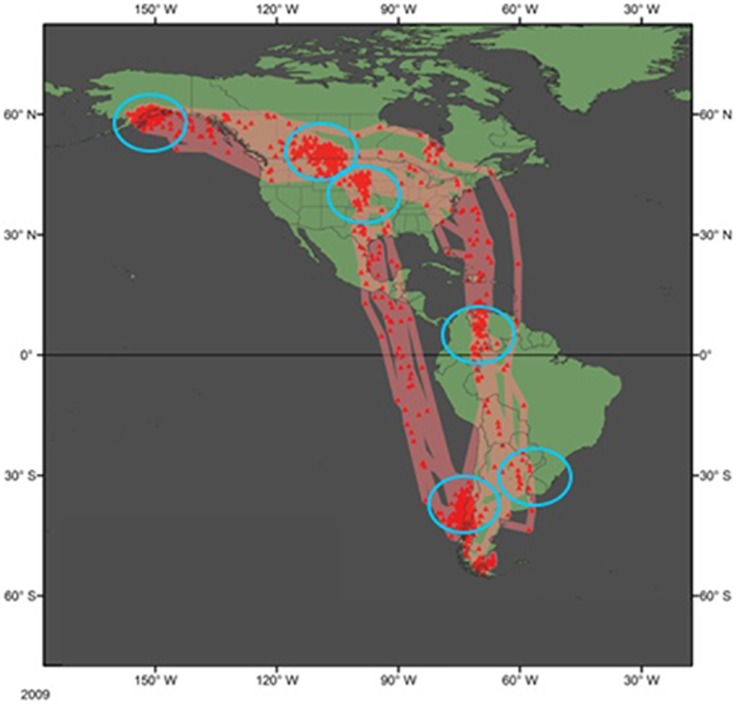
Map showing the migration routes of Hudsonian godwits breeding at Beluga River, Alaska. Twenty-six individuals were tracked across three years 2009–2012, though for ease of presentation this map only shows those from 2009–2010 (*n* = 12), using British Antarctic Survey Mk-14 geolocation-tracking devices. Each red triangle denotes the location of an individual on one day, but does not necessarily indicate that the individual stopped in that location. Each blue circle denotes a region in which the majority of godwits stopped and congregated in both years. From north to south, those regions are: Beluga River, Alaska (nesting site); central Saskatchewan (staging site southward migration); Rainwater Basin, Nebraska (staging site northward migration); Amazon Basin, Colómbia (stopover site southward migration); Buenos Aires Province, Argentina (stopover site southward migration); and Isla Chiloé, Chile (nonbreeding site). Note that the typical annual migratory route is a clock-wise loop.

Individual godwits repeatedly made non-stop flights of longer than 10,000 km and 7 days during their northbound migrations and flights of longer than 5 days and 6,500 km during their southbound migrations (Fig. 1). We found almost no inter-annual variation in migratory pathways. All but 2 of the 26 individuals stopped in the same suite of 6 regions each year — Beluga River (breeding site); central Saskatchewan (staging site during southward migration); Amazon Basin, Colómbia (stopover site during southward migration); Buenos Aires Province, Argentina (stopover site during southward migration); Isla Chiloé, Chile (nonbreeding site); and Rainwater Basin, Nebraska (staging site during northward migration). Beginning with departure from the breeding grounds in Beluga River each year, we were able to calculate mean annual population arrival and departure dates at each stopover site, as well as deviations in timing from these population averages for each individual (Table 1).

**Figure 2:**
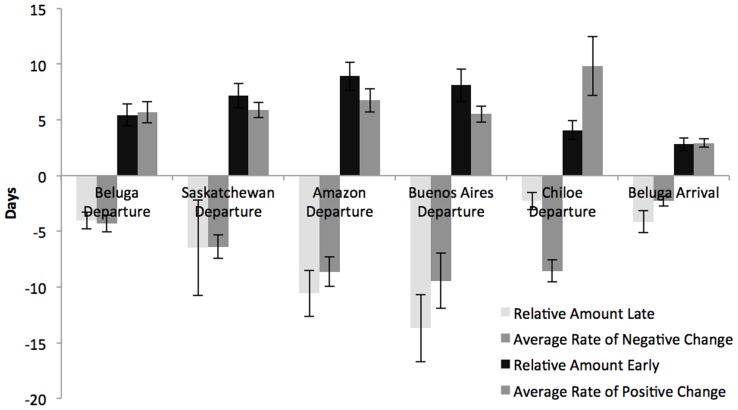
Average deviations in migration timing of 26 individual Hudsonian godwits tracked throughout their annual cycle, 2009–2012. Each bar denotes the average relative difference in timing of departure or rates of change for all individuals migrating either ahead or behind the population mean. Light gray and black bars represent average relative timing differences (see Step 1 in Table 1), while the two sets of darker gray bars represent average rates of change (see Step 2 in Table 1). Error bars represent standard errors.

Both the amount of variation in relative departure dates, and the magnitude of change in relative departure dates between stops, grew during the southward migration period (Fig. 2). However, these increases were not continuous, and individuals did not accumulate timing deviations throughout the entirety of their southward migration. Instead, while individual birds were either consistently earlier or later than population average departure times at successive stops, their rates of change from the population average were not correlated among stopover locations (Tables 2,3). Thus, while individual birds became progressively earlier or later during the course of their southward migration, additional deviations from population average timings did not necessarily occur at every stop. On average, by their departure from their last stopover site at Buenos Aires, late individuals were 13.7±3.0 (*n* = 17) days behind the population average, while early individuals were 8.5±1.5 (*n* = 27) days ahead of the population average.

**Figure 3:**
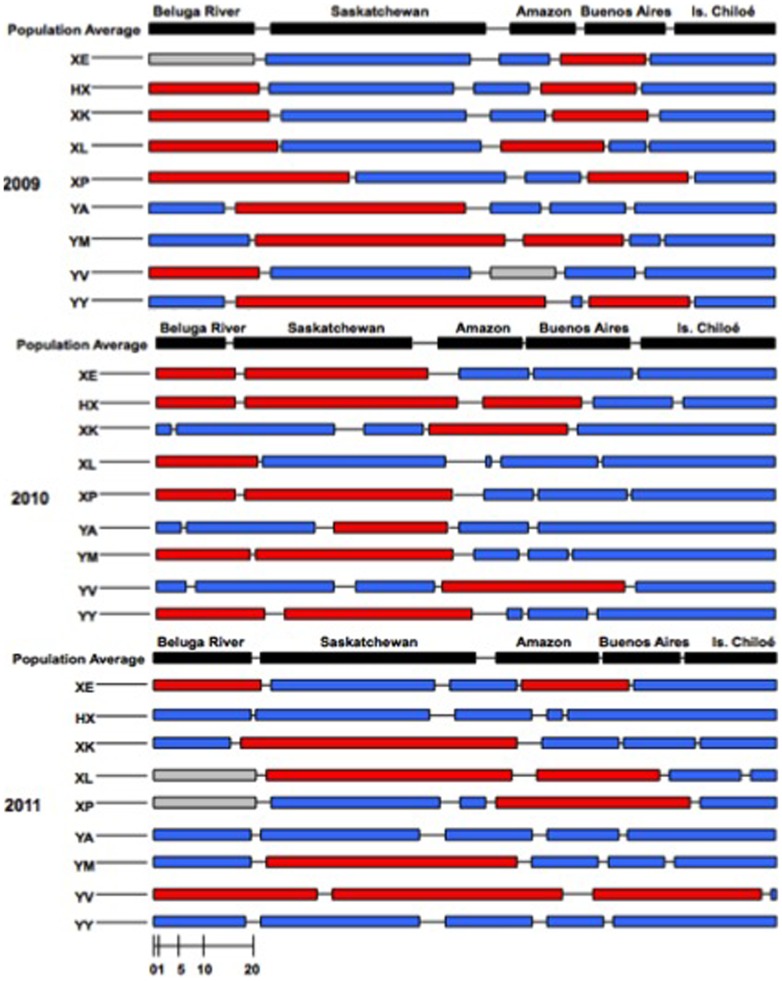
Deviations in timing from the population mean by nine individual Hudsonian godwits tracked during southward migration for three consecutive years, 2009–2011. Each bar denotes the number of days spent at a site and lines between bars the number of days spent traveling between sites. Red bars identify sites at which individuals became later relative to the population mean; blue bars those sites at which they became earlier relative to the population mean; gray bars those sites at which they neither became earlier nor later. The population average schedule for each year is shown in black.

Individuals did not continue to diverge from the population mean throughout an entire annual cycle because deviations disappeared during the period spent on Isla Chiloé (e.g., rate of change>relative timing deviation; Fig. 3), the godwits' southernmost destination, where they spent an average of 192±2 d (*n* = 46). The relative timing of an individual's arrival on Isla Chiloé and its rate of change were not correlated with those at its departure (Tables 2,3); one individual was even able to arrive 32 d after the mean arrival date and still depart 2 d before the mean departure date (individual “YJ,” 2010). While arrival within one year at Isla Chiloé could vary among all individuals by as much as 59 d (*μ* = 44±13 d, *n* = 3), departure could vary by as little as 7 d (*μ* = 9±1 d, *n* = 3). The average number of days by which individuals were behind or ahead of the population mean at departure were 2.3±0.8 d (*n* = 21) and 4.1±0.9 d (*n* = 16), for early and late individuals, respectively. Timing deviations and rates of changes did not grow in magnitude or accumulate during the northward migration — individuals were late by an average of 4.1±1.0 d (*n* = 14) or early by an average of 2.8±0.6 d (*n* = 22; Fig. 3) upon their arrival at their nesting site at Beluga River.

An individual's arrival date on the breeding grounds was uncorrelated with its departure date from Isla Chiloé and its deviation from population-average arrival dates at stopover sites in North America. Instead, an individual's arrival date on the breeding grounds was determined by the number of stops made en route (β = 2.19, *SE* = 0.51, *t* = 4.33, *P*<0.01) and the amount of time spent at those stops (β = 0.31, *SE* = 0.11, *t* = 2.85, *P*<0.01; Tables 2, 3), which should be viewed as proxies for the timing of departure from mid-continental stopover sites (see Materials and Methods).

Breeding success was correlated with neither the relative timing of arrival/departure nor rates of change from any period of the annual cycle (Table 4). The only variable that was related to breeding success was the number of nesting attempts undertaken by an individual (β = −0.41, *SE* = 0.18, *t* = −2.30, *P* = 0.03). All renesting attempts failed either during the incubation or chick phase (*n* = 22).

**Table 4:** Factors affecting the breeding success of Hudsonian godwits (2010–2012).

	Random Effects	
Variable	σ^2^	St. Dev.
Individual	0.00	0.00
Year	0.00	0.00
Residual	0.20	0.45

	Fixed Effects		
Variable	β	SE	*t*
Intercept	**2.57**	**1.01**	**2.56**
Beluga River Arrival T.D.	0.04	0.09	0.49
Chiloé Departure T.D.	−0.05	0.09	−0.52
Number of Stops	−0.16	0.32	−0.52
Average Stopover Duration	−0.04	0.04	−0.88
Number of Nests	**−0.41**	**0.19**	**−2.13**
Beluga River Arrival R.C.	−0.01	0.10	−0.13

Parameter estimates for a generalized linear mixed-effect model with a binomial error distribution predicting the breeding success of individual godwits (*n* = 26).

By and large, how early or late an individual was in one year, and the rate at which it departed from population-average timing of movements, was not related with its lateness in other years (Fig. 3; Tables 2,3). However, there were a small number of exceptions to this general pattern, with individual birds being consistent among years in: the relative timing of departure from the Amazon Basin stopover site (σ^2^ = 44.60, *SD* = 6.68, χ^2^ = 4.16, *P* = 0.04), rate of change at departure from the Amazon Basin stopover site (σ^2^ = 45.15, *SD* = 6.72, χ^2^ = 4.64, *P* = 0.03), and the relative timing of departure from Isla Chiloé (σ^2^ = 3.54, *SD* = 1.88, χ^2^ = 6.51, *P* = 0.01).

Potentially our data were biased by differences in the return rates of birds, such as lower return rates for birds that fell too far behind the population mean timing of movements. We found no suggestion of this bias, as individual return rates were high in all years (82.7±12.5% across all years). Additionally, carrying a data logger did not have a detectable effect on an adult's survival, as individuals carrying data loggers returned at higher rates than did those individuals carrying only alpha-alpha flags on their legs (83.5±10.0% vs. 80.9±16.5% respectively, across all years). A logistic regression including prior breeding success, number of nesting attempts, and accumulated lateness during the previous year did not explain the return rates of individuals carrying data loggers and no single variable had a statistically significant effect (Table 5). A mixed-model logistic regression of data from all banded adults also found that a model containing variables for prior breeding success, number of nesting attempts, and whether or not an individual was carrying a logger did not explain return rates better than a null model (ANOVA, df = 2, *P* = 1; Table 6).

**Table 5:** Factors affecting whether or not adult Hudsonian godwits carrying data loggers returned to the breeding grounds in the subsequent year, 2010–2012.

	Random Effects	
Variable	σ^2^	St. Dev.
Individual	2.43×10^−3^	0.49
Year	4.13×10^−10^	2.03×10^−5^

	Fixed Effects		
Variable	β	SE	*P*
Intercept	25.04	2.24×10^7^	1.00
Previous Breeding Success	−1.62	4.82×10^5^	1.00
Previous Number of Nesting Attempts	3.59	2.23×10^7^	1.00
Accumulated Lateness	0.14	100.61	0.99

Parameter estimates for a linear mixed-model predicting breeding ground return rates of individual godwits carrying data loggers. (*n* = 47).

**Table 6:** Factors affecting breeding ground return rates of all banded Hudsonian godwits, 2010–2011.

	Random Effects	
Variable	σ^2^	St. Dev.
Individual	50.03	7.09
Year	2.81×10^−12^	1.68×10^−6^

	Fixed Effects		
Variable	β	SE	*P*
Intercept	2.06	15.63	0.90
Previous Breeding Success	1.67	6.54	0.80
Previous Number of Nesting Attempts	3.06	15.19	0.84
Data Logger	−0.14	4.35	0.97

Parameter estimates for a mixed-effect logistic regression model predicting breeding ground return rates of individual godwits carrying alpha-alpha flags and data loggers. (*n* = 74).

## Discussion

This is one of the first studies to document how individuals of a migratory species accrue and dissipate a potential carry-over effect — delays in the timing of events — across their entire annual cycle, and to link these fluctuations to reproductive success and survival. We found that in spite of having one of the most extreme migrations of any migratory bird, returning godwits that migrated later than the population mean during one portion of their annual cycle did not remain behind for the entirety of their annual cycle, nor did they suffer reduced breeding success or survival. Authors of recent studies have marveled at the marathon distances traveled in non-stop migratory flights [19], [21], but also at the consistency of arrival and departure dates of individuals and the lack of apparent carry-over effects within some species [13], [22]. This combination suggests that strong selection has constrained the timing of movements as well as the selection of sites used during migratory stopovers [20]. The continued success of such a finely-tuned annual cycle further emphasizes the role played by habitat quality — every site used by these species must remain of sufficiently high quality to support individuals flying extreme distances, as well as recover from stresses accrued during previous flights and portions of the annual cycle [3], [4], [23]–[25]. Long-distance migratory birds thus provide a stark example of the potential for even slight changes in environmental conditions to have rapid and dramatic effects on population dynamics [26], [27].

### Deviations in Migratory Timing Across the Annual Cycle

We found that individual godwits that deviated from the population mean timing of movements during one portion of their annual cycle could continue to remain ahead or behind the population mean for as much as half of their annual cycle. For instance, some individuals that departed the breeding grounds on their southward migration later than average also arrived at Isla Chiloé on a later than average date. These individuals did not become progressively later at every subsequent stop in between these two events — there was no correlation between the rate of change exhibited by these individuals between sites — but their average deviation from mean timing did increase during the course of the southbound migration. Regardless of whether an individual arrived at Isla Chiloé ahead or behind the population mean, all individuals erased their overall deviation from mean timing during the nonbreeding period on Isla Chiloé: Departure dates from Isla Chiloé differed by as little as seven days among individuals within a year, even though arrival dates at the site could vary by as much as 59 days. Following their stay on Isla Chiloé, those individuals that did depart on their northward migration later than average did not necessarily arrive on the breeding grounds later than average, as arrival at the breeding grounds was both highly synchronous and correlated only with events that had occurred during migration in the mid-continental United States. Ultimately, breeding success was unaffected by the timing of arrival at the breeding grounds, or at any other location during the annual cycle. Instead, the only variable related to breeding success was the number of nests laid by an individual, likely because those individuals renesting after a depredation event were forced to raise their young in a resource poor environment (N.R. Senner unpubl. data)

Exhibiting significant flexibility in the timing of post-breeding migratory movements, but a highly canalized pre-breeding migration is not necessarily surprising. Both theoretical and empirical work have established the basis for this pattern [20], [28], [29]. It is nonetheless surprising and highly unusual to document the complete disconnection between these timing deviations and measured fitness and survival among individuals, especially given the apparent ubiquity of carry-over effects amongst migratory taxa [5]. Given that we expected carry-over effects to be a particularly strong driver of godwit migration timing and reproductive success, our findings suggest the need for a reassessment of which species are most likely to suffer from carry-over effects and, more broadly, what phenomena should be considered carry-over effects.

### What is Significant About Migratory Timing Deviations?

Harrison et al. [5] define carry-over effects as the events and processes occurring in one season that cause an individual to transition to the next season in a different condition, such that subsequent performance is affected. Because we do not have physical observations of the birds and their conditions outside of the breeding season, we cannot unequivocally refute the hypothesis that carry-over effects affected those godwits consistently migrating later than the population mean during one migratory period. We argue, though, that carry-over effects are not affecting late individuals, as these timing deviations carried no apparent fitness consequences in either the form of reproductive failure or lowered survival. Nor, however, are these timing deviations simply aspects of individually unique migratory schedules. If that were the case, both of our sets of models would have consistently identified “individual” as a significant random effect (see Materials and Methods), implying individual consistency in timing across years. We found, instead, that different individuals in different years deviated from mean migratory timing and we hypothesize that this reflects the stability and abundance of food resources throughout the period and the strong selection acting upon godwits on their flights between stopover sites.

During their southbound migration, godwits make three stops between southcentral Alaska and Isla Chiloé — central Saskatchewan, Canada; the northwestern Amazon Basin of Colómbia and Brazil; and the coast of the Buenos Aires province of Argentina. Flights to and among stops average three, five, two, and one day(s) respectively. While these pale in comparison to the seven day, 10,000+ km flight which godwits undertake during their northward migration, or the nine day, 11,000+ km flight that they have been recorded undertaking with Bar-tailed Godwits, *Limosa lapponica*, en route to New Zealand [30], they are not without their potential perils: The flight between Saskatchewan and the Amazon Basin totals more than 6,500 km and involves a significant ocean crossing during the peak of hurricane season [31]. Similarly, the flight from Buenos Aires to Isla Chiloé involves a crossing of the Andes Mountains, a major barrier to avian movements in other taxa [32]. There is thus likely strong selection acting upon godwits to adequately prepare for these flights, as there may be little opportunity for emergency stopovers — and we recorded none — if an individual depletes its resources or other conditions mid-flight become inclement [18], [19], [33]–[36].

If there is strong selection acting to insure successful non-stop, long-distance flights, but little apparent selection on migration timing on southward migration, resource abundance (to fuel such long flights) and stability must remain high throughout the southward migration period [27]. All surviving godwits, no matter the timing of their movements or order of arrival at a site, must be able to find adequate resources to both successfully complete the next stage of their migration and do so without compromising their condition to such an extent that it affects their subsequent flight. If this were not the case, we would expect to see evidence that timing deviations would remain throughout the year or that late godwits would exhibit unusually low survival [37].

Are godwits thus impervious to carry-over effects? The answer is likely no, but three key points should be kept in mind. First, it is possible that we did not identify any carry-over effects because our study did not include a year during which we would expect carry-over effects to have been generated [38]. However, our study did encompass both a series of years with high numbers of North Atlantic hurricanes [39], which affected other long-distance migratory birds (F. Smith pers. comm.), and one of the most severe droughts to ever affect the mid-continental United States [40]. Given the severity of these conditions, we might reasonably expect to have identified carry-over effects if they were present. Second, regardless of why individuals exhibit migratory timing deviations, Isla Chiloé appears to play a pivotal role in resynchronizing the timing of movements of all godwits — individuals that arrived at Isla Chiloé as much as 32 days later than average could still depart the site ahead of the population mean. Great flexibility in the timing and duration of those activities carried out on the nonbreeding grounds (i.e., molt) is not unique among godwits [22], but suggests in all cases the overriding importance of a high quality nonbreeding site. Without such a high quality nonbreeding site, it is easy to imagine the timing deviations that developed during southward migration growing unabated throughout the nonbreeding season and into the northward migration when migration timing does appear to be under strong selection [41]. Third, we may not observe carry-over effects among godwits, because all godwits suffering from carry-over effects may perish during migratory flights. The fact that godwit survival is uncorrelated with accumulated delays during the previous year suggests that transitioning between events or seasons in poor condition simply may not be possible and that weak individuals are rapidly selected against. However, because godwits also continue to experience high inter-annual survival, the suite of sites currently used by godwits must be healthy enough to support a stable population [42].

### Conservation Implications

The godwit annual cycle, more so even than the annual cycles of most other species, appears to be predicated on the existence of a string of sites with high quality, super-abundant resources that remain readily available over a long period of time and are predictable from year to year. Such sites are highly uncommon and a reduction in the quality of any of these sites could have severe impacts on the ability of the species to complete its migration [43]. The example of *rufa* red knots, *Calidris canutus rufa*, is telling in this respect [26]. In Delaware Bay, a reduction in the quality of the final stopover site of red knots before a long, non-stop flight has resulted in a dramatic population decline, concomitant with the appearance of carry-over effects. For godwits, which employ even longer non-stop flights, carry-over effects may potentially never appear and there may be no intermediary between their current situation, with an apparently stable population, and rapid population declines. Thus, in our study system, decreasing the quality of any single site, but especially the nonbreeding areas on Isla Chiloé could have significant detrimental impacts on the entire annual cycle and population health of Hudsonian Godwits. Prioritizing the protection of these sites may disproportionately contribute to the continued viability of godwit populations [14], [27].
